# Effects of Oliceridine Versus Sufentanil on Postoperative Recovery Quality During Hysteroscopy Under Laryngeal Mask Airway Anesthesia: Protocol for a Single-Blind and Randomized Controlled Trial

**DOI:** 10.2196/84521

**Published:** 2026-01-02

**Authors:** YuHang Shou, JianSheng Luo, XianJie Zhang, WenHu Zhai, Jia Han

**Affiliations:** 1 Department of Anesthesiology Deyang People’s Hospital Deyang China; 2 Department of Clinical Medicine School of Medical and Life Sciences Chengdu University of Traditional Chinese Medicine Chengdu China

**Keywords:** oliceridine, hysteroscopy, quality of recovery, opioid-related adverse events, sufentanil, general anesthesia

## Abstract

**Background:**

Hysteroscopy, the gold standard for diagnosing and treating intrauterine pathologies, has shown substantial increase in its adoption in clinical practice. Nevertheless, early postoperative pain and opioid-related adverse effects remain critical determinants of recovery quality. Oliceridine—a novel G protein-biased μ-opioid receptor agonist—demonstrates an improved therapeutic range over conventional opioids in preliminary studies.

**Objective:**

This study aims to evaluate whether oliceridine enhances recovery quality while reducing opioid-related complications compared to sufentanil in patients undergoing hysteroscopy under general anesthesia.

**Methods:**

This single-center randomized controlled trial will enroll 120 patients undergoing hysteroscopy under general anesthesia with 1:1 randomization to sufentanil- or oliceridine-based analgesia. The primary outcome is early recovery quality assessed by the 15-item Quality of Recovery scale at 24 hours after the surgery, while secondary outcomes include hemodynamic fluctuations during induction, total intraoperative opioid consumption and supplemental bolus frequency, proportion requiring vasoactive agents, incidence of respiratory depression in postanesthesia care unit, postoperative extubation time, opioid-related adverse events within 24 hours, and Visual Analog Scale pain scores at 30 minutes, 4 hours, 8 hours, and 24 hours postextubation.

**Results:**

This study received approval from the Medical Ethics Committee of Deyang People’s Hospital, Deyang, China, on April 16, 2025 (approval 2025-03-009-K01). Participant recruitment is anticipated to be completed by December 2025. Data analysis, manuscript preparation, and submission for publication are expected to be completed by February 2026.

**Conclusions:**

The successful completion of this trial will generate evidence regarding whether oliceridine enhances recovery quality while reducing opioid-related complications compared to sufentanil in patients undergoing hysteroscopy under general anesthesia.

**Trial Registration:**

Chinese Clinical Trial Registry ChiCTR2500104024; https://www.chictr.org.cn/showproj.html?proj=275501

**International Registered Report Identifier (IRRID):**

DERR1-10.2196/84521

## Introduction

Hysteroscopy is currently recognized as the gold standard for diagnosing and treating intrauterine pathologies, having revolutionized gynecological diagnostics and operative interventions. This modality enables both diagnostic evaluations and surgical treatments through a minimally invasive approach, characterized by rapid postoperative recovery. Advancements in hysteroscopic technologies and instrumentation have substantially increased its adoption across diverse patient populations [1,2]. Pain remains a leading cause of procedural failure during hysteroscopy. To optimize surgical conditions and enhance satisfaction for both patients and surgeons, hysteroscopic procedures in China are predominantly performed under general anesthesia. A nationwide survey revealed that 63.8% of the hysteroscopic surgeries used anesthesia in 2021, underscoring its critical role in contemporary practice [3]. European data corroborate this pattern, with a retrospective analysis confirming that a proportion of hysteroscopic procedures also necessitate general anesthesia for successful completion [4].

To date, no robust evidence exists to define optimal anesthesia protocols for pain management in hysteroscopy, and standardized approaches remain underexplored. For hysteroscopic procedures anticipated to be lengthy or involving significant tissue trauma, the 2020 Chinese Expert Consensus on Anesthesia Management recommends general anesthesia with laryngeal mask airway (LMA) to ensure procedural tolerance [5]. Current clinical practice predominantly uses conventional opioids such as sufentanil for intraoperative analgesia. However, sufentanil administration is associated with multiple opioid-related adverse effects, including nausea, vomiting, excessive sedation, respiratory depression, drug dependence, and opioid-induced hyperalgesia, which collectively compromise early postoperative recovery [6].

Oliceridine is a novel G protein-biased μ-opioid receptor agonist that selectively activates G protein signaling while minimizing β-arrestin recruitment. This pharmacological profile confers a mechanistic advantage: selectively engaging intracellular analgesic pathways while avoiding those mediating adverse effects [7,8]. As such, oliceridine has garnered emerging clinical interest for hysteroscopic analgesia.

The 15-item Quality of Recovery scale (QoR-15) is a multidimensional construct evaluating patient-centered recovery, encompassing physical, physiological, psychological, social, and functional domains [9,10]. The QoR-15—validated for reliability and clinical utility—quantifies recovery across 5 dimensions: physical comfort, emotional status, psychological support, independence, and pain experience. Postoperative pain intensity, nausea/vomiting incidence, and opioid consumption profiles constitute key determinants of patient recovery quality [11,12].

Therefore, this study uses QoR-15 as the primary endpoint to evaluate oliceridine's efficacy within LMA-based general anesthesia for hysteroscopy. We aim to determine whether oliceridine enhances recovery quality while reducing opioid-related complications compared to sufentanil in patients undergoing hysteroscopy under general anesthesia.

## Methods

### Study Design

**Figure 1:**
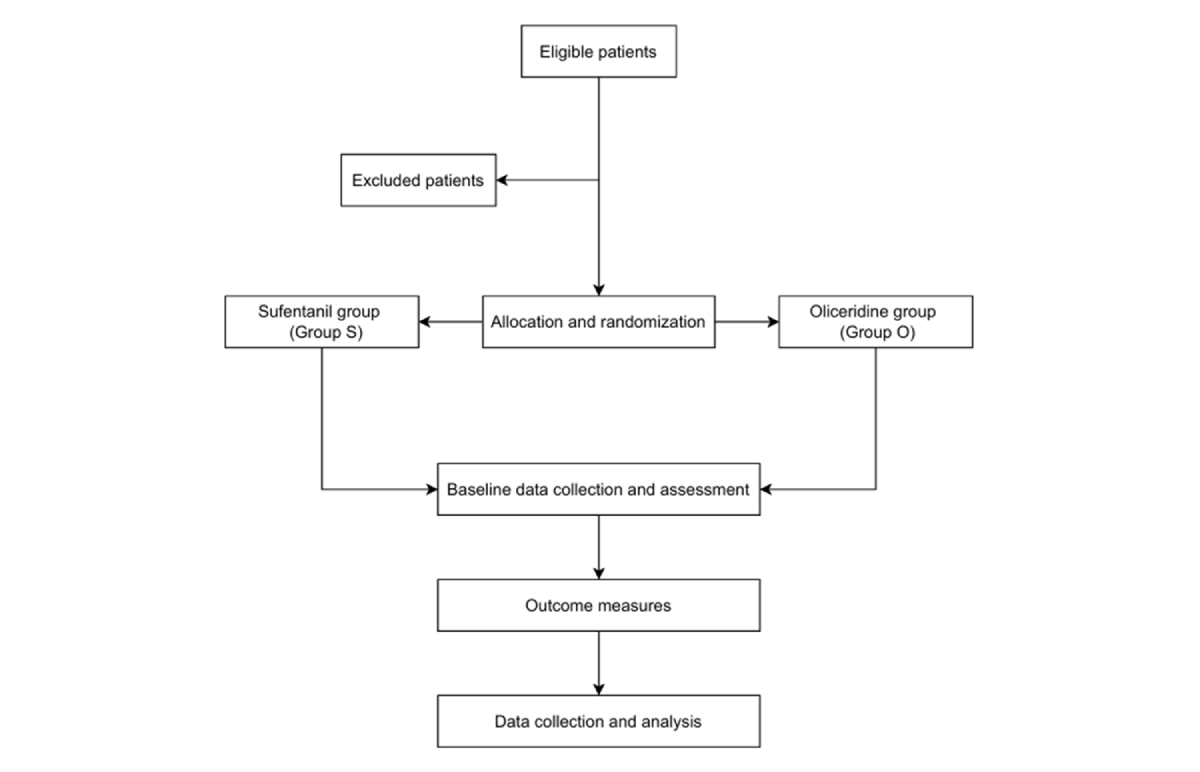
Flowchart for this study.

This prospective, single-center, single-blind, randomized controlled study will be conducted at Deyang People's Hospital, Deyang, China. The protocol received ethical approval from the institutional ethics committee (2025-03-K01) and was registered at the Chinese Clinical Trial Registry on June 10, 2025. Written informed consent will be obtained from all participants or their legal guardians. Eligible patients will be randomly allocated to either the sufentanil (S) group or oliceridine (O) group. The participant recruitment timeline is shown in Figure 1. A time schedule for the enrollment, interventions, and assessments is detailed in Table 1.

**Table 1:** The study schedule of the enrollment, interventions, and assessments.

Timepoint		Enrollment phase (day 1)	Allocation (before surgery)	Surgery (during anesthesia)	Follow-up (within 24 h after surgery)
**Enrollment**					
	Eligibility screening	✓			
	Informed consent	✓			
	Allocation		✓		
**Interventions**					
	Sufentanil			✓	
	Oliceridine			✓	
**Assessments**					
	Baseline data	✓			
	Primary outcome				✓
	Secondary outcome			✓	✓

### Eligibility Criteria

Inclusion criteria are as follows: (1) patients scheduled for hysteroscopic surgery under LMA general anesthesia who were assessed by physicians as unable to tolerate the pain stimuli associated with surgical manipulation, (2) patients aged 18 to 65 years, (3) patients with American Society of Anesthesiologists physical status I-III, and (4) patients who voluntarily sign informed consent.

Exclusion criteria are as follows: (1) patients with American Society of Anesthesiologists physical status IV or higher (indicating severe systemic disease, functional incapacity, including but not limited to New York Heart Association class III or higher heart failure, myocardial ischemia, severe reduction in cardiac ejection fraction, third-degree atrioventricular block, stage 3 hypertension, respiratory failure, severe chronic obstructive pulmonary disease, severe pulmonary dysfunction, or decompensated hepatic or renal insufficiency); (2) patients with known allergy to any component of the investigational drugs or contraindications to their use; (3) patients unable to cooperate (eg, psychiatric disorders, anticipated difficulty with neuropsychological assessment, or communication barriers due to pronunciation or dialect); (4) patients with a history of alcohol or substance abuse; (5) patients with concurrent participation or participation in another clinical trial within 12 weeks prior to this trial; (6) patients with BMI>30 kg/m²; (7) patients unable to comprehend the study procedures or refusal to participate; and (8) patients with any condition deemed by the investigator to pose an unacceptable risk or to contraindicate participation, such as anticipated difficult airway management.

### Randomization and Blinding

Block randomization with block sizes of 4 or 6 will be performed using a computer-generated sequence. An independent researcher not involved in the trial will prepare sequentially numbered, opaque, sealed envelopes containing group assignments. Participants will be allocated in a 1:1 ratio to either the experimental or control group by opening these envelopes in a sequential order at the time of enrollment. Allocation cards will be sealed inside opaque envelopes to ensure concealment. The study medications (oliceridine or sufentanil) will be prepared by a dedicated research nurse as visually identical clear solutions in 10-mL syringes. These prepared syringes will then be provided to the anesthesiologist, who will remain unaware of the treatment allocation. Blinding will be maintained for the participants, the anesthesiologist, and the outcome assessors throughout the trial to minimize assessment bias. The blinding will be broken only in the event of severe adverse events. Study participants, their legally authorized representatives, the anesthesiologist, and outcome assessors will be blinded to group allocation throughout the trial. Unblinding the allocation is generally not required per protocol. Emergency unblinding will be performed exclusively to determine rescue medications for critical adverse events or during other medical emergencies

### Interventions

#### Explanation for the Choice of Comparators

This study evaluates the impact of oliceridine (group O) on early postoperative recovery quality in patients undergoing hysteroscopic surgery under general anesthesia. The sufentanil-based regimen (group S) serves as the comparator because of its status as the conventional analgesic approach for such procedures in current clinical practice.

Both groups will receive identical anesthesia induction and maintenance protocols but with divergent analgesic regimens. Group O will be administered oliceridine at a dose of 60 μg/kg (diluted to 10 mL with normal saline) as a slow intravenous bolus for induction. Supplemental doses of 30 μg/kg will be provided if intraoperative analgesia is deemed inadequate, defined as mean arterial pressure and/or heart rate exceeding 20% above baseline values. Group S will receive sufentanil at a dose of 0.3 μg/kg (similarly diluted to 10 mL) as an induction bolus, with supplemental doses of 0.15 μg/kg administered under the same hemodynamic criteria.

#### Anesthesia Protocol

Upon arrival in the operating room, standard monitoring, including pulse oximetry, invasive radial arterial blood pressure, and electrocardiography will be initiated. Thirty minutes prior to induction, the attending anesthesiologist will administer intravenous penehyclidine hydrochloride (0.01 mg/kg) and ondansetron (4 mg). Anesthesia induction will begin with a slow intravenous bolus of either sufentanil or oliceridine according to group assignment. One minute later, propofol (2 mg/kg) and cisatracurium (0.15 mg/kg) will be administered. After satisfactory neuromuscular blockade is achieved, an LMA will be inserted. Mechanical ventilation will be initiated with a tidal volume of 6-8 mL/kg predicted body weight and positive end-expiratory pressure of 5 cmH₂O. Anesthesia will be maintained using a continuous infusion of propofol (4-12 mg/kg/h), with supplemental opioids administered based on hemodynamic criteria (mean arterial pressure or heart rate >20% above baseline). The propofol infusion rate will be titrated to maintain a bispectral index value between 40 and 60. Neuromuscular blockade will be maintained with intermittent boluses of cisatracurium (one-third of the induction dose). If reversal is required, neostigmine (0.02 mg/kg) and atropine (0.01 mg/kg) will be administered. After surgery, all patients will be transferred to the postanesthesia care unit.

#### Criteria for Discontinuing or Modifying Allocated Interventions

The criteria for discontinuing are as follows: (1) severe anesthesia/surgical complications (eg, anaphylaxis, shock, cardiac arrest, malignant arrhythmias, aspiration requiring intervention, malignant hyperthermia), (2) protocol deviation in anesthesia/surgical technique, (3) withdrawal of consent, (4) anticipated difficult airway, and (5) failed LMA placement.

#### Strategies to Improve Adherence to Interventions

The principal investigator will conduct preoperative evaluations against inclusion/exclusion criteria one day prior to surgery. During informed consent, the investigator will detail the study procedures to participants and legally authorized representatives, outlining participant responsibilities.

#### Relevant Concomitant Care Permitted or Prohibited During the Trial

The provision of additional care during the trial followed institutional protocols.

### Outcomes

The primary outcome is defined as the quality of postoperative recovery at 24 hours after surgery and will be assessed using QoR-15 (score range: 0-150; higher scores indicate better recovery). Secondary efficacy endpoints are (1) hemodynamic changes during induction (mean arterial pressure/heart rate at T0 [pre-drug], T1 [pre-LMA], T2 [post-LMA immediate], T3 [5-min post-LMA]); (2) total intraoperative opioid consumption and supplemental bolus frequency; (3) proportion requiring vasoactive agents; (4) incidence of postanesthesia care unit respiratory depression (defined as respiratory rate <6 bpm, end-tidal carbon dioxide amplitude change >50 mm Hg or <30 mm Hg, or waveform loss >20 s); (5) time to extubation; (6) opioid-related adverse events within 24 hours; and (7) Visual Analog Scale pain scores at 30 minutes, 4 hours, 8 hours, and 24 hours postextubation.

### Data Collection and Management

Baseline data will include demographic characteristics (age, sex, BMI), the preoperative QoR-15 score, and comorbidities with diagnostic verification. Intraoperative records will include vital signs, anesthetic agents/dosages, surgical duration, and proportion requiring vasopressor administration. In the postanesthesia care unit, time to extubation, early postoperative Visual Analog Scale pain scores, and incidence of respiratory depression will be documented. Postoperative recovery quality will be assessed at 24 hours by using QoR-15, with 24-hour adverse events systematically recorded. Predefined subgroup analyses stratified by age will be conducted for primary and secondary outcomes, applying identical statistical approaches as specified for the main analyses.

Source data will be documented using standardized case report forms. To minimize data entry errors, independent dual-entry verification will be performed. Physical records will be stored securely in locked cabinets with restricted access within the Department of Anesthesiology. Electronic data will be hosted on encrypted, cloud-based platforms.

### Data Reporting Guidelines

The SPIRIT (Standard Protocol Items: Recommendations for Interventional Trials) reporting guidelines will be used for this paper (Multimedia Appendix 1).

### Sample Size

An initial retrospective review of 10 patients who underwent hysteroscopy under sufentanil-based general anesthesia resulted in a mean QoR-15 score of 129.8 (SD 4.92). Based on published literature, the minimal clinically important difference for QoR-15 is 6 [13]. We hypothesize that oliceridine will improve the postoperative QoR-15 score by this minimal clinically important difference (6 points). Sample size was calculated using PASS 15 software with the following parameters: a standard deviation of 11.5 [13], a 2-sided α of 0.05, and 90% power. The calculation indicated that 48 patients are required per group. To account for an estimated 20% attrition rate, the total sample size was set at 120 patients (60 per group).

### Recruitment Plan

Based on institutional surgical volume projections, patient recruitment is scheduled to commence in July 2025 and is anticipated to be completed within a 5-month period to enroll the target cohort of 120 participants. Eligible patients will be identified primarily through active screening of institutional surgical schedules for planned hysteroscopies. Preoperative screening for eligible candidates will be conducted by the investigators. Written informed consent will be obtained from all participants or their legally authorized representatives either before or on the day of surgery. The consent process, approved by the institutional review board, ensures comprehensive disclosure of the potential benefits, risks, and alternative treatments. Documentation will confirm that all participants have fully understood the information before providing their signature.

### Statistical Analysis

The primary analysis will be conducted on a modified intention-to-treat population, defined as all randomized participants who receive the study drug (oliceridine or sufentanil) at anesthesia induction. The per-protocol population will consist of modified intention-to-treat participants who complete the 24-hour follow-up assessment without major protocol deviations, serving as the population for supportive analyses. All participants who receive the study drug will be included in the safety analysis set.

Primary/secondary outcomes will be analyzed using SPSS software (version 27.0; IBM Corp), with continuous variables first assessed for normality via Kolmogorov-Smirnov testing; normally distributed data will be expressed as mean (SD), nonnormal data as median (IQR), and categorical variables as counts (%). Quantitative between-group comparisons will employ independent-samples *t* tests or Mann-Whitney *U* tests, while qualitative comparisons will use chi-square, continuity-corrected chi-square, or Fisher exact tests, with all tests being 2-tailed (statistical significance threshold: *P*<.05).

### Methods to Handle Protocol Nonadherence and Any Statistical Methods to Handle Missing Data

Prior to enrollment, comprehensive study details will be disclosed to potential participants, with written informed consent obtained from patients and their legally authorized representatives. Investigators may discontinue participation for substantial protocol deviations (eg, nonattendance at scheduled visits), initiating replacement recruitment according to the randomization sequence.

### Plans to Promote Participant Retention and Complete Follow-Up

Investigators will maintain ongoing communication with the participants throughout the trial to address concerns while ensuring protocol adherence. Participant involvement may be discontinued by investigators in cases of nonadherence to scheduled visits or substantial protocol deviations, with all collected data excluded from the final analysis per intention-to-treat principles.

### Ethical Considerations

This study received approval from the institutional ethics committee of Deyang People's Hospital (approval 2025-03-009-K01). All procedures strictly adhered to legislative and institutional requirements, with no involvement of vulnerable populations. Eligible patients will undergo preoperative screening. Written informed consent will be obtained from all participants or their legal guardians prior to or on the day of surgery. We ensure comprehensive disclosure of potential benefits and risks, with full participant comprehension verified before consent signing. Participants will be informed that participation is voluntary and that they may withdraw at any time without penalty. Each participant will receive ¥100 (US $14.12) as compensation. All investigators adhere strictly to confidentiality protocols and ethical standards throughout the trial. Personally identifiable data will be maintained with maximized confidentiality. Confidential information undergoes secure processing/storage protocols, with exclusively anonymized datasets shared for analysis.

### Dissemination Plans

Study findings will be submitted for publication in peer-reviewed anesthesiology journals. Access to trial data and protocols is exclusively restricted to the trial leader. No personnel may access participant data without documented prior approval from the principal investigator.

## Results

The first patient was enrolled on July 11, 2025. As of September 2025, a total of 55 participants has been enrolled. Data analysis, manuscript preparation, and submission for publication are expected to take place throughout the first quarter of 2026.

## Discussion

### Anticipated Findings

Hysteroscopy serves as a minimally invasive modality for diagnosing and treating intrauterine pathologies, with indispensable roles in managing endometrial polyps, abnormal uterine bleeding, intrauterine adhesions, uterine septa, endometrial hyperplasia, and early-stage endometrial cancer [14-16]. Procedures requiring prolonged operative time or large instrumentation necessitate anesthesia in operating room settings [17]. In China, LMA-based general anesthesia is currently a prevalent approach for hysteroscopic surgery [18]. Conventional analgesic protocols predominantly use traditional opioids such as sufentanil. However, these agents frequently induce respiratory/circulatory depression, nausea/vomiting, excessive sedation, immunosuppression, opioid-induced hyperalgesia, constipation, and abdominal distension [19-22]. Although effective for anesthesia, these complications prolong hospitalization, increase perioperative management complexity, and compromise postoperative recovery quality. Consequently, reducing opioid-related adverse events is crucial for enhancing recovery.

Oliceridine is a synthetic μ-opioid receptor agonist distinguished from conventional opioids by its G protein-biased selectivity, which mediates potent analgesia while substantially reducing β-arrestin recruitment—a signaling pathway strongly associated with opioid-related adverse events. In contrast, traditional opioids (eg, morphine, fentanyl) nonselectively activate both G protein and β-arrestin pathways upon μ-receptor binding [23,24]. Clinical trial evidence confirms that oliceridine significantly reduces nausea, vomiting, and respiratory complications compared to morphine [25-28]. Therefore, we postulate that this mechanistic advantage of oliceridine may similarly confer clinical benefits when compared with sufentanil.

QoR-15 was selected to assess postoperative recovery quality as a validated patient-reported outcome measure evaluating 5 key recovery dimensions following anesthesia and surgery. Its established reliability and practical clinical utility underpin widespread adoption [11]. The Chinese version has been formally validated, demonstrating robust reliability, validity, and user-friendliness [29]. These properties support its extensive application in clinical trials for postoperative recovery assessment.

### Limitations

No prior studies have established whether oliceridine’s mechanistic advantages translate to enhanced recovery quality in hysteroscopic surgery under general anesthesia. However, there are a number of potential limitations in the study design: (1) single-center design—though protocol standardization ensures internal validity, generalizability may be limited; (2) exclusive focus on 24-hour recovery without long-term follow-up; and (3) exclusion of high-risk populations (BMI>30 kg/m²), who exhibit heightened susceptibility to opioid-related complications and may theoretically derive the greatest benefit from oliceridine.

## Appendix

Multimedia Appendix 1 SPIRIT checklist.

## Abbreviations

LMA: laryngeal mask airway
QoR-15: 15-item Quality of Recovery scale
SPIRIT: Standard Protocol Items: Recommendations for Interventional Trials

## Data Availability

Deidentified participant data generated in this study will be made accessible upon reasonable request to the corresponding author following paper publication.
